# In Silico Characterization of the Interaction between the PBP2a “Decoy” Protein of Resistant *Staphylococcus aureus* and the Monomeric Units of Eudragit E-100 and Poly(Maleic Acid-*alt*-Octadecene) Polymers

**DOI:** 10.3390/polym13142320

**Published:** 2021-07-15

**Authors:** Yamil Liscano, Ana Amú, Astrid González, Jose Oñate-Garzón, Constain H. Salamanca

**Affiliations:** 1 Grupo de Investigación en Química y Biotecnología (QUIBIO), Facultad de Ciencias Básicas, Universidad Santiago de Cali, Calle 5 No. 62-00, Cali 760035, Colombia; yamil.liscano00@usc.edu.co (Y.L.); ana.amu00@usc.edu.co (A.A.); astrid.gonzalez00@usc.edu.co (A.G.); 2 Departamento de Farmacia, Facultad de Ciencias Farmacéuticas y de Alimentos, Universidad de Antioquia, Calle 67 No. 53-108, Medellín 050010, Colombia; 3 Laboratorio de Diseño y Formulación de Productos Químicos y Derivados, Departamento de Ciencias Farmacéuticas, Facultad de Ciencias Naturales, Universidad ICESI, Calle 18 No. 122-135, Cali 760035, Colombia

**Keywords:** ampicillin, docking, eudragit E-100, mrsa strain, PBP2a protein, poly(maleic acid-*alt*-octadecene)

## Abstract

Antimicrobial treatment alternatives for methicillin-resistant *Staphylococcus aureus* (MRSA) are increasingly limited. MRSA strains are resistant to methicillin due to the formation of β-lactamase enzymes, as well as the acquisition of the mecA gene, which encodes the penicillin-binding protein (PBP2a) that reduces the affinity for β-lactam drugs. Previous studies have shown that the use of ampicillin-loaded nanoparticles can improve antimicrobial activity on resistant *S. aureus* strains. However, the biological mechanism of this effect has not yet been properly elucidated. Therefore, this short communication focused on characterizing the in silico interactions of the PBP2a membrane receptor protein from *S. aureus* against the monomeric units of two polymeric materials previously used in the development of different nanoparticles loaded with ampicillin. Such polymers correspond to Eudragit E-100 chloride (EuCl) and the sodium salt of poly(maleic acid-*alt*-octadecene) (PAM-18Na). For this, molecular coupling studies were carried out in the active site of the PBP2a protein with the monomeric units of both polymers in neutral and ionized form, as well as with ampicillin antibiotic (model β-lactam drug). The results showed that ampicillin, as well as the monomeric units of EuCl and PAM18Na, described a slight binding free energy to the PBPa2 protein. In addition, it was found that the amino acids of the active site of the PBPa2 protein have interactions of different types and intensities, suggesting, in turn, different forms of protein–substrate coupling.

## 1. Introduction

β-lactam antibiotics, such as ampicillin, are broad-spectrum bactericidal drugs used for the treatment of infectious diseases caused by several bacterial organisms [1]. Among these, *Staphylococcus aureus* is considered one of the most significant intrahospital pathogen because it can colonize ventilated patients and burned units, thereby contributing to airborne dissemination via droplets between medical staff and colonized⁄infected patients [2], leading to the acquisition of multiple nosocomial infections, and some strains may even produce toxins that provoke both primary and secondary bacteremia, gastroenteritis, intra-abdominal⁄pelvic abscesses, toxic shock syndrome, osteomyelitis and scalded skin syndrome [2,3]. Regarding the pharmacological mechanism of ampicillin, it is based on its union with PBP proteins (Penicillin-Binding Proteins), which are enzymes anchored on the cell membrane that contribute to the construction of peptidoglycan as a component of the cell wall. In this way, ampicillin inhibits the last stage of synthesis of the cell wall of microorganisms [4]. However, this inhibition capability has been lost over time, leading to what is widely known as *antibiotic resistance* as a result of the emergence of the isozyme PBP2a in MRSA strains, which exhibits a low affinity for β-lactam antibiotics [5,6]. Such resistance can be acquired for several reasons, where bacterial evolutionary processes and the inappropriate use of antibiotics stand out [7], such as self-medication, which is conjunctural with the lack of knowledge of the antibiotic dose and the time of treatment, variables that contribute to the development of resistance mechanisms [8].

In relation to the generation of resistance of *S. aureus* regarding to β-lactam antibiotics, it has been widely described that it depends on two factors: (i) The production of specialized enzymes (β-lactamases) that degrade the β-lactam ring of these antibiotics and (ii) the acquisition of the *mecA* gene that encodes the PBP2a protein. The activity of this transpeptidase protein, which acts as a decoy for β-lactam drugs, is regulated by allosterium at a site 60 Å distant from the active site, where crosslinking of the cell wall takes place [9,10,11].

This problem has highlighted the need to continue developing new and diverse alternatives, such as the synthesis of new antibiotics or compounds that mimic the β-lactam ring such as clavulanic acid and sulbactam [12], as well as the use of nanotechnology of polymeric particles. This new field has been growing considerably in the last two decades, where the antimicrobial activity of various types of nanoparticles (NPs) loaded with antibiotics has been evaluated. Among these, polymer–drug nanocomplexes [13], polymer-coated nanocapsules [14] and self-assembly systems (polymeric micelles and polymer-coated liposomes) [15] are the most commonly employed.

**Figure 1:**
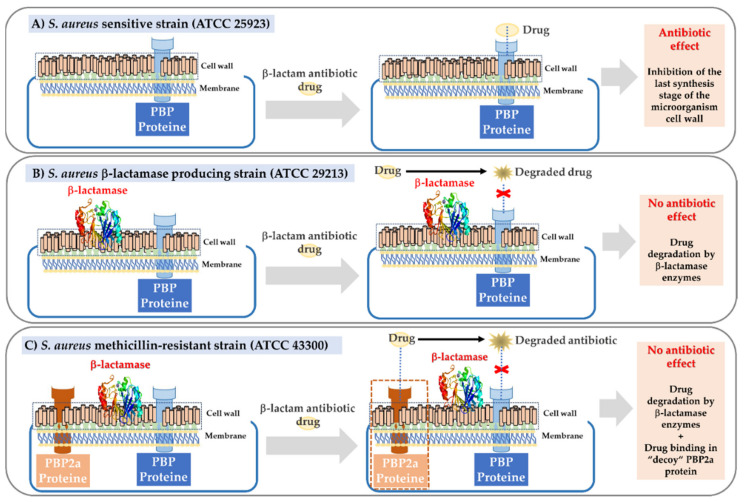
Scheme of the pharmacological mechanisms of β-lactam drugs on *S. aureus* strains with different degrees of resistance. (**A**) Sensitive strain. (**B**) β-lactamase producing strain. (**C**) Methicillin-resistant strain with a dual mechanism (β-lactamase and PBP2a “decoy” protein production).

In accordance with the aforementioned, some of our studies have focused on evaluating the effect of several ampicillin-loaded NPs against *S. aureus* strains with different degrees of antibiotic resistance (ATCC 25923-sensitive strain, ATCC 29213-β-lactamase producing strain and ATCC 43300-encoding the *mecA* gene and β-lactamase producing strain). The ATCC 29213 strain is characterized by a resistance mechanism based on the formation of β-lactamase enzymes, while the ATCC 43300 strain, which also produces these enzymes, has an extra mechanism of encoding, the PBP2a “decoy” protein, converting it into the most resistant model strain (Figure 1).

**Figure 2:**
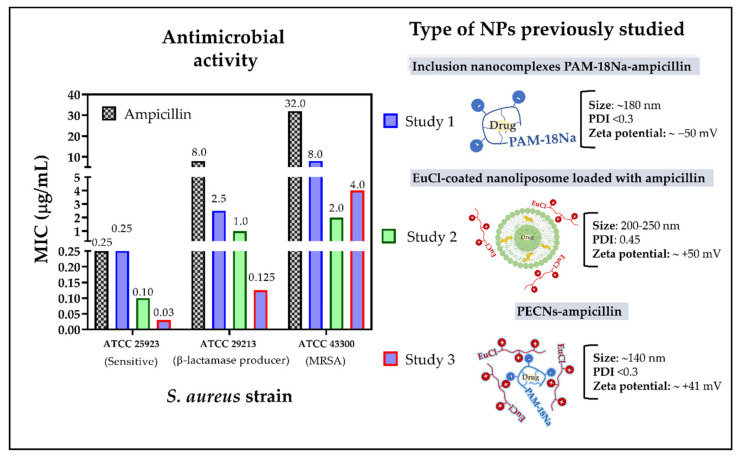
Results of the minimum inhibitory concentrations (MIC) of different NPs developed in previous studies on *S. aureus* strains with different degrees of resistance.

Accordingly, our first study focused on evaluating the antimicrobial effect of the inclusion nanocomplexes formed between ampicillin and the sodium salt of poly(maleic acid-*alt*-octadecane) (PAM-18Na). This study demonstrated a 3- and 4-fold increase in antimicrobial activity in resistant *S. aureus* strains ATCC 29213 (β-lactamase producer) and ATCC 43300 (MRSA), respectively, when ampicillin was nanocomplexed. Likewise, this study also showed that these inclusion nanocomplexes can considerably decrease the degradation of ampicillin against β-lacatamase enzymes [16]. The second study evaluated the effect of nanoliposomes coated with the cationic polymer Eudragit E-100 chloride (EuCl) and loaded with ampicillin, finding an increase in the antimicrobial activity of 8- and 16-fold against the ATCC 29213 and ATCC 43300 strains, respectively [17]. In contrast, the third study assessed different polyelectrolyte complex nanoparticles (PECNs) formed between PAM-18Na and EuCl polymers loaded with ampicillin. In this case, a moderate increase in antimicrobial activity was found on each of the resistant *S. aureus* strains [18]. Therefore, the results described in the three previous studies suggested that depending on the type of nanoparticle there is a particular antimicrobial effect (Figure 2). Nevertheless, none of these studies could clarify whether the observed antimicrobial results were due to the NPs providing a protective effect to the drug against β-lactamase enzymes or preventing them from binding to the PBP2a “decoy” protein or both. Hence, this study is focused on evaluating the in silico interactions of the PBP2a protein against the monomeric units of two polymeric materials previously employed in the development of ampicillin-loaded NPs, allowing us to describe the way in such NPs increase the antimicrobial effect in *S. aureus* resistant strains.

## 2. Materials and Methods

### 2.1. Obtaining 3D Ligands

3D structure of the monomeric units was obtained using the online version of the Chemdraw software (https://chemdrawdirect.perkinelmer.cloud/js/sample/index.html (accessed on 20 June 2021)). On the other hand, ampicillin was obtained from the PubChem database (ID 107676). In order to obtain the PDB files for molecular docking, the structures of the monomeric units and ampicillin were optimized using the Avogadro software version 1.2 (https://avogadro.cc/ (accessed on 20 June 2021)), applying fields of force MMFF94 (Merch Molecular Force Field) [19] and GAFF (General AMBER Force Field) [20], respectively.

### 2.2. PBP2a Proteine

The structure of the penicillin-binding protein (PBP2a) of the methicillin-resistant *S. aureus* strain 27 r was acquired from the Protein Data Bank (PDB) database with ID 1VQQ. Subsequently, the structure was validated with PROSA (https://prosa.services.came.sbg.ac.at/prosa. Php (accessed on 21 June 2021)) and Molprobity (http://molprobity.biochem.duke.edu/ (accessed on 21 June 2021))

### 2.3. Docking Molecular

Molecular docking between the receptor (PBP2a) and the ligands (PAM-18Na, Eudragit E-100 and ampicillin) were carried out using PyMOL [21]. The receptor treatment consisted of the water molecules’ extraction, and the addition of Kollmann charges and hydrogen atoms to optimize the hydrogen bonds [22]. Ligand preparation was carried out by adding hydrogen atoms and Gasteiger charges. Likewise, the grid coordinates were obtained using the previously prepared ligand and protein by means of CB-Dock online tool (http://cao.labshare.cn/cbdock/ (accessed on 21 June 2021)). In contrast, the Discovery Visualizer Study Software (http://accelrys.com (accessed on 21 June 2021)) was used to analyze ligand–receptor interactions in order to find the amino acids that interact with the ligands.

## 3. Results and Discussion

**Figure 3:**
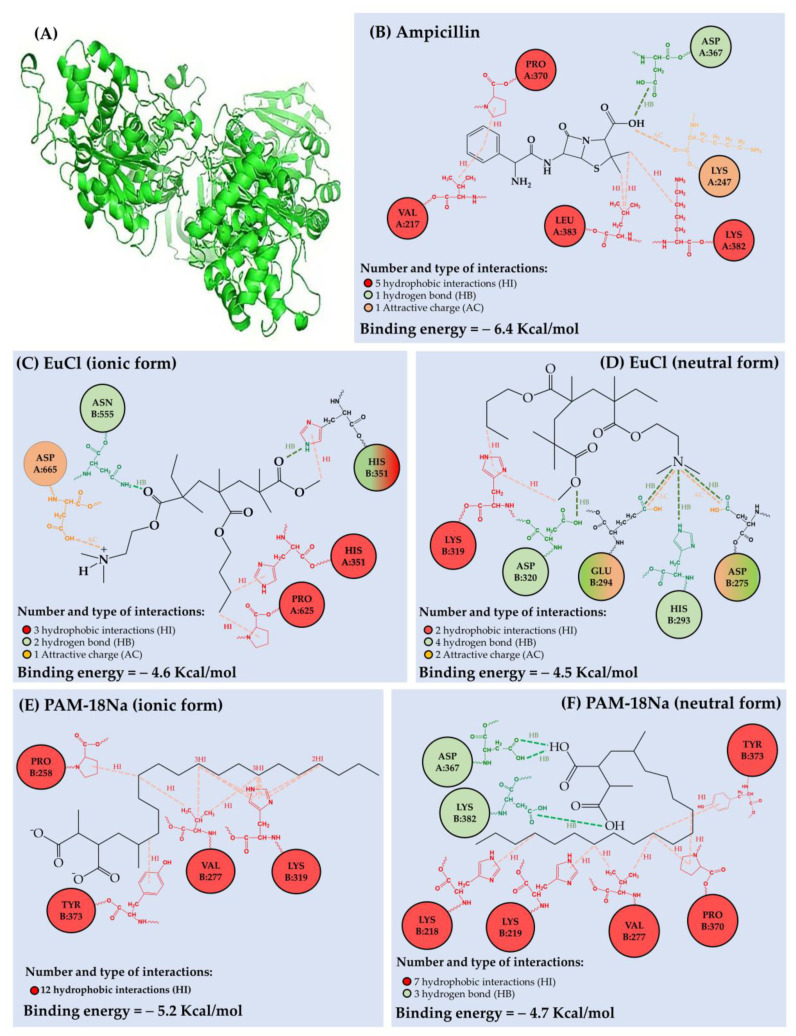
(**A**) Optimized three-dimensional structure of the PBPa2 protein. Scheme of the interactions and coupling free energy between (**B**) PBPa2-ampicillin, (**C**) PBPa2-EuCl (ionic form), (**D**) PBPa2-EuCl (neutral form), (**E**) PBPa2-PAM18Na (ionic form), (**F**) PBPa2-PAM18Na (neutral form).

PBP2a protein optimization, as well as the results of interactions and free energy of binding between protein active sites and ampicillin, EuCl and PAM18Na substrates are presented in Figure 3. The number and type of interactions between the different ligands and PBP2a is summarized in Table 1. The results showed that ampicillin has several interaction positions with the PBP2a protein, corresponding to five hydrophobic interactions (HI), one hydrogen bond (HB) and one attractive charge (AC) interaction with a total binding free energy of −6.4 kcal/mol (Figure 3B). Regarding HI, they occurred between the ampicillin phenyl-substituent and the alkyl chain of VAL A:217 and the pyrrolidine ring of PRO A:370. Other three HI were generated between the β-lactam methylenes of ampicillin and the butyl-amino and isobutyl of LYS A:382 and LEU A:383, respectively. Furthermore, it was found that ampicillin exhibited a HB with ASP A:367, as well as an AC interaction (ion–dipole) with LYS A:382. These result suggests that ampicillin and the protein amino acids are in an acid–base thermodynamic equilibrium, leading to a slight protein–substrate affinity. However, these interactions are not sufficient to form a favorable bond between the β-lactam ring of ampicillin and the nucleophilic serine of the active site of PBP2a [23].

Regarding the interactions generated between PBP2a protein and EuCl monomeric units, they are lower than those described by ampicillin and also depend on the polymeric ionization degree. In the case of the EuCl ionic form (Figure 3C), this polymer formed three HI, one HB and one AC interaction with a total binding free energy of −4.6 Kcal/mol. The HI were generated between the polymeric methyl and butylmethacrylate groups and the imidazole and pyrrolidine rings of HIS A:351 and PRO A:625., respectively. In contrast, the HB and AC interactions were produced between the dimethylaminoethyl (DMAE) group of EuCl and ASN B:555 and ASP A:665 amino acids, respectively. In relation to EuCl neutral form (Figure 3D), it produced two HI, four HB and two AC interactions with a total binding free energy of −4.5 Kcal/mol. In this case, HI occurred between the imidazole ring of LYS B:319 and the polymeric substituents methyl and butyl methacrylate. Concerning the HB interactions, these were generated between the methylmethacrylate group of the polymer and the carboxylic acid of ASP B:320. Likewise, the nitrogen of the polymeric DMAE group generated three extra HB with HIS B:293, GLU B:294 and ASP B:275. Similarly, GLU B:294 and ASP B:275 amino acids generated AC interactions (ion–dipole) with DMAE group of EuCl. These results suggest that the free energy of the binding between the PBP2a protein and the EuCl polymer is weak, where the polymeric ionization degree does not considerably affect the interaction strength, but it does affect the site of the interaction with the protein.

On the other hand, the results of the interactions generated between the PBP2a protein and the PAM-18Na monomer units showed a low intensity of interaction, which depends on the polymer ionization degree. In the case of the PAM-18Na ionic form (Figure 3E), it was found that it presented a total binding free energy of −5.2 Kcal/mol. Such energy is exclusively due to twelve hydrophobic interactions given between the polymeric alkyl chain and the pyrrolidine, phenyl, isopropyl and butyl-amino groups of the protein amino acids PRO B:258, TYR B:373, VAL B:277 and LYS B:319, respectively. Conversely, the PAM-18Na neutral form (Figure 3F) described seven HI and three HB with a total binding free energy of −4.7 Kcal/mol. The HI were generated between the polymeric alkyl chain and the pyrrolidine, phenyl, isopropyl and butyl-amino groups of PRO B:370, TYR B:373, VAL B:279 and LYS B:218–219 amino acids, respectively. In contrast, the HB were formed between the carboxylic acids of PAM-18Na polymer and ASP A:367 LYS A:382 amino acids. Such a result is very interesting, since ampicillin also binds to both amino acids (ASP A:367 LYS A:382). However, it is important to highlight that both polymeric materials also have a high conformational dynamism and with this, the sites of interaction with the PBP2a protein can be modified. Therefore, when the polymer has a high ionization degree, there is an increase in electrostatic repulsion in the polymer backbone, leading to extended conformation. In contrast, when the polymer has a low ionization degree, a hydrophobic effect occurs with the polymeric side alkyl chains, leading to a coiled conformation [24,25].

Finally, all these results suggest that EuCl and PAM-18Na polymers previously employed in the development of ampicillin-loaded NPs and that showed an increase in antimicrobial activity on resistant *S. aureus* strains, do not have an significant interaction with the PBP2a protein. In this way, it can be established that the increases in the antimicrobial activity observed in such studies mainly depend on the protection of ampicillin by NPs against b-lactamase enzymes and not on the interaction with the PBP2a protein. However, the hypothesis that such polymers may have affinities with other components of the outer bacterial wall of *S. aureus* strains ATCC 43300 is not completely discarded. Foxley et al. revealed that branched polyethyleneamine bound to lipoteichoic acid in the cell wall of MRSA strains, increasing the antibacterial activity of ampicillin, even surpassing that of vancomycin [26].

## 4. Conclusions

The EuCl and PAM-18Na polymers showed a moderate interaction with the PBP2a protein and different types of interactions were revealed. Therefore, the antimicrobial activity of ampicillin-loaded Nps formed by such polymers and described in our previous studies could be attributed mainly to a protective effect of ampicillin against β-lactamase enzymes.

## Figures and Tables

**Table 1:** Number, type of interaction and binding energy between the different ligands and the PBP2a protein.

Molecule	Binding Energy (kcal/mol)	HI	HB	EI
Ampicillin	−6.4	5	1	1
Eudragit (ionic form)	−4.6	3	2	1
Eudragit (neutral form)	−4.5	2	4	2
PAM18 (ionic form)	−5.2	12	0	0
PAM18 (neutral form)	−4.7	7	3	0

HI = Hydrophobic Interactions; HB = Hydrogen Bonds; EI = Electrostatic Interactions.

## Data Availability

Not applicable.
